# Alteration of Ileal lncRNAs After Duodenal–Jejunal Bypass Is Associated With Regulation of Lipid and Amino Acid Metabolism

**DOI:** 10.3389/fphys.2022.836918

**Published:** 2022-04-07

**Authors:** Yongjun Liang, Minghua Yu, Yueqian Wang, Mengyi Li, Zhongtao Zhang, Zhengdong Qiao, Peng Zhang

**Affiliations:** ^1^ Center for Medical Research and Innovation, Shanghai Pudong Hospital, Fudan University Pudong Medical Center, Shanghai, China; ^2^ Shanghai Key Laboratory of Vascular Lesions Regulation and Remodeling, Shanghai Pudong Hospital, Fudan University Pudong Medical Center, Shanghai, China; ^3^ Department of Surgery, Capital Medical University Beijing Friendship Hospital, Beijing, China

**Keywords:** duodenal–jejunal bypass, lncRNAs, ileum, lipid metabolism, amino acid metabolism

## Abstract

Metabolic and bariatric surgery (MBS) can generate a drastic shift of coding and noncoding RNA expression patterns in the gastrointestinal system, which triggers organ function remodeling and may induce type 2 diabetes (T2D) remission. Our previous studies have demonstrated that the altered expression profiles of duodenal and jejunal long noncoding RNAs (lncRNAs) after the duodenal–jejunal bypass (DJB), an investigational procedure and research tool of MBS, can improve glycemic control by modulating the entero-pancreatic axis and gut–brain axis, respectively. As an indiscerptible part of the intestine, the ileal lncRNA expression signatures after DJB and the critical pathways associated with postoperative correction of the impaired metabolism need to be investigated too. High-fat diet-induced diabetic mice were randomly assigned into two groups receiving either DJB or sham surgery. Compared to the sham group, 1,425 dysregulated ileal lncRNAs and 552 co-expressed mRNAs were identified in the DJB group. Bioinformatics analysis of the differently expressed mRNAs and predicted target genes or transcriptional factors indicated that the dysregulated ileal lncRNAs were associated with lipid and amino acid metabolism-related pathways. Moreover, a series of lncRNAs and their potential target mRNAs, especially NONMMUT040618, *Pxmp4*, *Pnpla3*, and *Car5a*, were identified on the pathway. In conclusion**,** DJB can induce remarkable alteration of ileal lncRNA and mRNA expression. The role of the ileum in DJB tends to re-establish the energy homeostasis by regulating the lipid and amino acid metabolism.

## Introduction

The prevalence of type 2 diabetes mellitus (T2DM) has been soaring globally. According to the International Diabetes Federation’s Diabetes Atlas 7th edition, about 415 million people in the world are suffering from diabetes, and the projected total number of T2DM patients will reach 642 million by 2035 eventually (Ogurtsova et al., 2017). T2DM is a complex metabolic homeostasis disorder and mainly manifested as hyperglycemia, which, if uncontrolled, can potentially lead to an increased risk of microvascular- and macrovascular-related complications (Lai et al., 2014).

Metabolic and bariatric surgery (MBS), such as Roux-en-Y gastric bypass (RYGB) and sleeve gastrectomy (SG) procedures, emerged as a weight-reduction therapy originally and has been clinically shown to be able to induce long-term remission of T2DM (Carlsson et al., 2012; Mingrone et al., 2012; Chang et al., 2014), to reduce cardiovascular risk factors (Sjostrom et al., 2012), and to decrease overall mortality (Sjostrom et al., 2007; Chang et al., 2014) in addition to drastic weight loss. During the past decades, a great deal of research efforts has been invested in the investigation of the mechanisms of MBS. Some related factors, such as weight reduction, caloric restriction, change in bile acid secretion, fibroblast growth factor 19 (*FGF19*), gut microbiome, and gastrointestinal (GI) hormonal changes, were proposed. However, none of the factors can explain all biological and physiological changes after MBS. So far, the exact underlying mechanism remains unknown.

All MBS procedures are involved with the anatomical alteration of the GI tract, which seems to be the origin of all the biological and physiological benefits induced by MBS. Therefore, in order to better understand the mechanisms of MBS, it is crucial to illustrate the functional role of the GI tract in metabolic modulation, although the GI tract was traditionally considered the digestive system. During the past few years, our team carried out a series of studies to investigate the role of each GI segment in metabolic regulation using diabetic animal models and gene microarray analysis. Whole transcriptome analysis of long noncoding RNAs (lncRNAs) and co-expressed mRNAs is a fundamental and powerful tool to understand the functional alteration of organs caused by surgical intervention. Once considered to be “transcriptional noise”, lncRNAs are currently defined as transcripts longer than 200 nucleotides that lack protein-coding capability but regulate almost all biological processes (Mercer and Mattick, 2013). Under normal circumstances, serving as signals, decoys, guides, and scaffolds, lncRNAs maintain physiological homeostasis. While under the pathological conditions, abnormal expression of lncRNAs participates in the human disease development (Kwok and Tay, 2017). For instance, peripheral insulin resistance (Zhu et al., 2016) and inflammatory diabetes complications (Reddy et al., 2014) are precisely governed by lncRNAs.

From our previous studies, we have revealed that 1) gastric volume reduction is essential for T2DM remission after RYGB (Zhang et al., 2016); 2) drastic changes in lncRNA and mRNA expression profiles of the duodenum induced by duodenal exclusion attenuate inflammation and initiate insulin secretion via the entero-pancreatic axis (Liang et al., 2017); and 3) alteration of the jejunal Roux limb lncRNA and mRNA expression pattern triggers both neuromodulation and endocrine-related pathways and participates in T2DM remission after duodenal–jejunal bypass (DJB) via the gut–brain axis (Liang et al., 2018). Apparently, beyond the traditional cognition, the duodenum and jejunum are more like metabolic regulators cross-talking with peripheral organs and executing surgical information, rather than solely digestive and absorptive organs. Reasonably, considering the altered anatomical structure and the faster passage of meal chyme after surgery, the ileum should also be involved in metabolic reconstruction, although its role has not been characterized yet. As an investigational procedure and research tool of MBS, DJB only causes anatomical changes in the intestine, which can rule out interference from other factors. Therefore, in the present study, we utilized a rodent T2DM model to elucidate the molecular mechanism on how the ileum modulates metabolic regulation after DJB.

## Materials and Methods

### Animal Model and Phenotyping

All procedures of this study were approved (NO. 2021-DS-M-09) by the Institutional Animal Care and Utilization Committee of the Fudan University Pudong Medical Center. A cohort of male C57BL/6 mice was received from Shanghai Slyke Laboratory Animal Corporation (Shanghai, China). After 1-week acclimatization, the mice started to undergo high-fat diet (HFD, 5.24 kcal/g with kcal percentages 60% fat, 20% protein, and 20% carbohydrate, Cat. #D12492, Research Diets Inc., New Brunswick, NJ, United States) at an age of 6 weeks for 12 weeks. At the end of the 12-week HFD, according to the weight (≈50 g), fasting blood glucose (FBG≥11 mmol/L), and impaired oral glucose tolerance test (OGTT), eight animals with typical diabetic phenotypes were selected and randomized into DJB or sham groups based upon the procedure received. The DJB procedure was performed with the 5-cm biliopancreatic limb and 4-cm Roux limb under general anesthesia, and the sham group only underwent the same general anesthesia and laparotomy but without intestinal transection and rerouting. Please refer to our previously published studies for the details of DJB and sham procedures (Liang et al., 2017; Liang et al., 2018). Another five age-matched mice served as the chow diet control without undergoing any surgical procedures. The mice were sacrificed two weeks after surgery, and the ileum was collected for RNA analysis. The body weight and FBG were measured after 6 h of fasting at 0 week (before initiation of HFD), 12 weeks (12 weeks after HFD), and 14 weeks (2 weeks after DJB/sham procedure). According to the manual, the plasma insulin levels were measured using enzyme-linked immunosorbent assay kits (Cat. #10-1247-01, Mercodia, Uppsala, Sweden). The OGTT was conducted after a 12-h overnight fast. After a 20% (w/v) glucose solution challenge at a dose of 2 g/kg weight, blood glucose levels were measured at different time points. The area under curve of the glucose excursions during OGTT was calculated.

### Ileal RNA Preparation and Microarray Analysis

Ileal RNA isolation, quality control, labeling, and hybridization methods have been described in detail previously (Liang et al., 2017; Liang et al., 2018). In brief, after hybridization, the “flag” value of each gene is calculated and classified into three levels of A, M, and P according to the signal strength. The genes with low hybridization signals (A or M) in both groups have been filtered out. Then, lncRNAs and mRNAs showing statistically significant differences in expression between the two groups were identified through *p* value/false-discovery (FDR) rate filtering. Hierarchical clustering was performed to demonstrate the expression patterns of critical lncRNAs and mRNAs. Significant differentially expressed lncRNA and mRNA were identified through fold change ≥2 and *p* < 0.05 and displayed by the scatter plot and volcano plot.

### Bioinformatic Analysis

The Gene Ontology (GO) and Kyoto Encyclopedia of Genes and Genomes (KEGG) pathway analysis were performed based on the function of lncRNA-co-expressed mRNAs. The target genes of lncRNAs were predicted via cis- or trans-regulatory patterns. Cis-regulated target gene prediction used the University of California Santa Cruz genome Browser to identify the potential targets located within a 10-kb window upstream or downstream of lncRNAs. Trans-regulated target gene prediction used Basic Local Alignment Search Tool software to screen the mRNAs that had sequences complementary to the lncRNAs, followed by identification of trans-acting target genes with RNAplex software (University of Vienna, Vienna, Austria). The potential transcription factors (TFs) targeting lncRNAs were predicted using TFSearch (National Institute of Advanced Industrial Science and Technology, Tsukuba, Japan). Co-expression and interaction networks were constructed to identify the core lncRNAs or mRNAs. The Pearson’s correlation coefficient (PCC) was used to calculate the expression correlation of lncRNAs and mRNAs. The pairs with PCC values >0.99 were selected as linkages in the network using Cytoscape 3.8.2 software (Agilent and IBS).

### Microarray Validation

Real-time PCR was used to confirm microarray results with a randomly selected subset of differentially expressed lncRNAs and mRNAs. The primers used in this study are shown in the Supplementary Material. Total RNAs were extracted using TRIzol reagent (Cat. #15596018, Invitrogen, Grand Island, NY, United States) and were reverse transcribed to cDNA with the PrimeScript RT Reagent Kit (Cat. #RR047A, Takara, Dalian, China). Real-time PCR was carried out in triplicate for each sample, with GAPDH as an internal reference, using PowerUp™ SYBR™ Green PCR Master Mix (Cat. #A25742, Life Technologies, Grand Island, NY, United States). Probe specificity was confirmed by melting curve analysis, and the relative expression of the genes was calculated using the 2^−△△Ct^ method.

### Statistical Analysis

Data were expressed as means ± SEM. Statistical analysis was performed using Student’s *t*-test to compare the differences between DJB and sham groups with SPSS software (Version 22.0; SPSS Inc., Chicago, IL, United States). Statistical significance was set at *p* < 0.05.

## Results

### DJB Improved Diabetic Phenotypes

**FIGURE 1:**
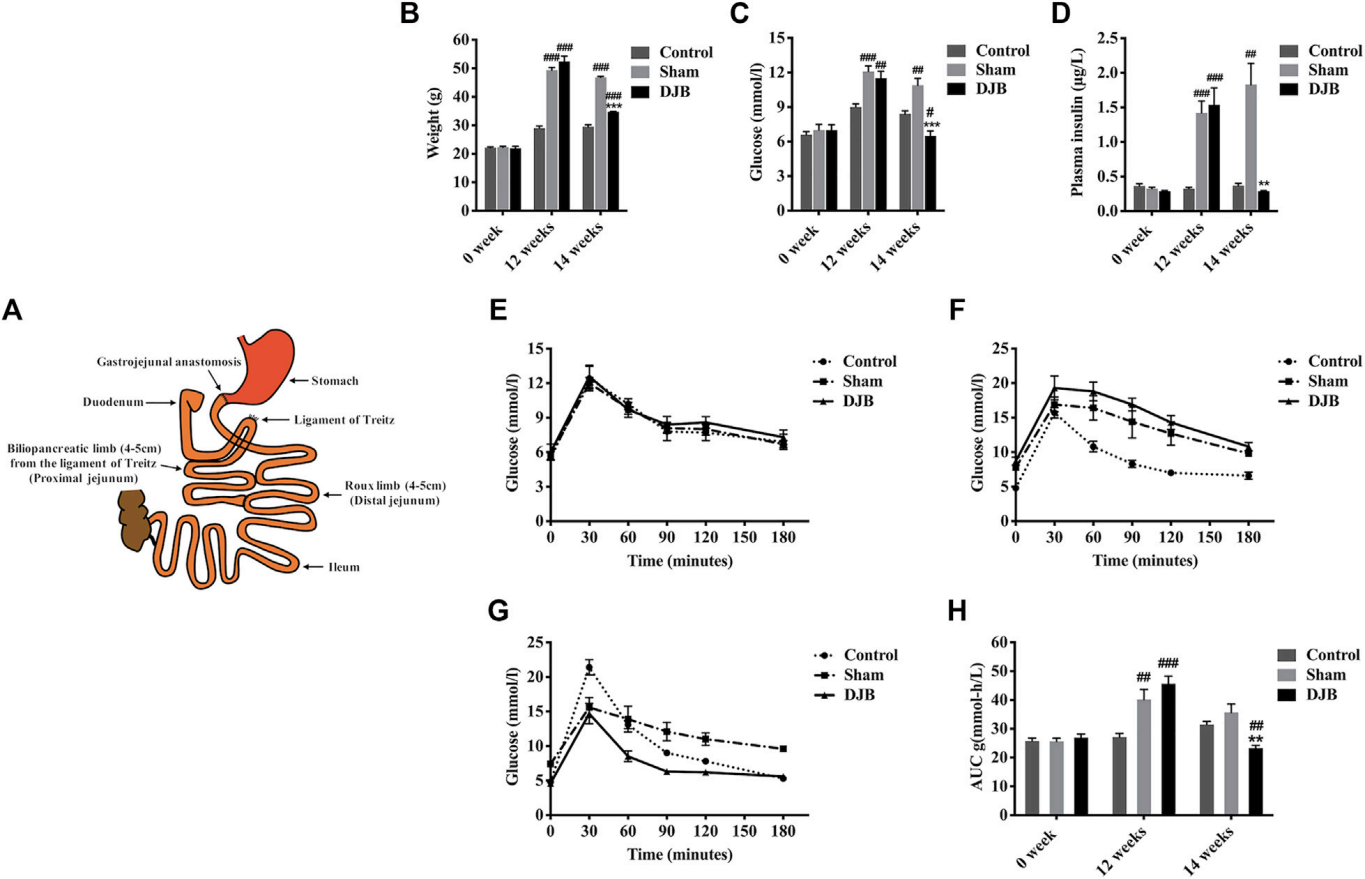
Characterization of the HFD-induced T2DM mouse model and surgical outcomes. Schematic diagram of DJB surgery **(A)**. Weight **(B)**, FBG **(C)**, plasma insulin **(D)**, OGTT before HFD induction **(E)**, OGTT at 12 weeks after HFD induction and before DJB **(F)**, 14 weeks on HFD and 2 weeks after DJB surgery **(G)**, and area under the curve (AUC) of OGTT glucose excursion **(H)**. All values are shown as mean ± SEM. Statistical differences were analyzed by the Student’s *t*-test. * For DJB group versus sham group, # for DJB or sham group versus control group (*p <0 .05, **p <0 .01, ***p <0 .001, #*p* <0 .05, ##*p* <0 .01, ###*p* <0 .001).

DJB surgery performed in our study is shown in Figure 1A. Before HFD induction (week 0), the baseline values of weight, FBG, plasma insulin, and oral glucose tolerance test (OGTT) of mice in different groups were almost identical (Figures 1B–E,H). After 12-week HFD, the mice in both DJB and sham groups successfully established diabetic phenotypes with elevated body weight, hyperglycemia, and hyperinsulinemia (Figures 1B–D,F,H). Compared to the sham group, two weeks after surgery (week 14), all metabolic parameters in the DJB group were rapidly improved and approached to the level of the chow diet control group (Figures 1B–D,G,H). Overall, the success of diabetic mouse model establishment and surgical procedure ensured the reliability of the following ileal microarray analysis.

### DJB Altered Ileal Transcriptome

**FIGURE 2:**
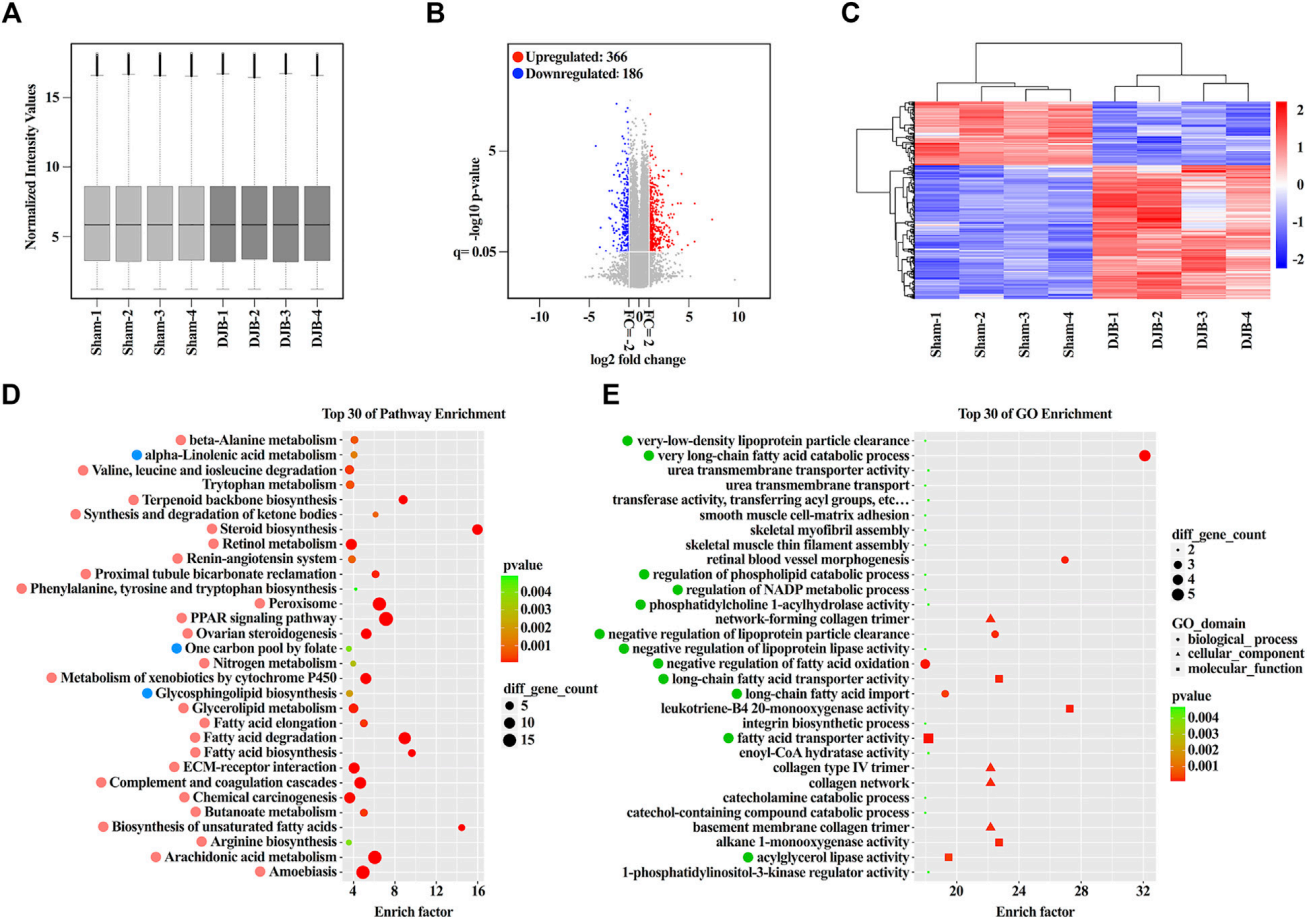
Overview and bioinformatic analysis of lncRNA-co-expressed mRNA profiles. **(A)** Box-plot showing the normalization of all samples. **(B)** Volcano plot of differentially expressed mRNAs between DJB and sham groups. **(C)** Heat map of mRNAs showed hierarchical clustering of differentially expressed mRNAs in DJB and sham mice (fold change ≥2, *p* < 0.05). Red and blue, respectively, represent high and low relative expression. **(D)** Top 30 significantly enriched pathways ranked with an enrichment score (*p* < 0.05). Up- and downregulated pathways are marked with soft red and soft blue, respectively. **(E)** Top 30 significantly enriched GO items (*p* < 0.05). The important items are marked with green.

The transcriptomes of eight ileal samples randomly selected from the DJB or sham group were investigated and found that their intensity values were nearly identical (Figure 2A). Compared to the sham group, 366 up- and 186 downregulated mRNAs were identified in the DJB group (Figure 2B). The number of mRNAs with different multiples was calculated (Supplementary Table S1), and the top 10 dysregulated mRNAs are listed in Supplementary Table S2. As a member of the phospholipase A2 enzyme family, *Pla2g4c* usually contributes to lipid metabolism by hydrolyzing glycerophospholipids to produce free fatty acids and lysophospholipids. While another gene *Cpvl*, a novel serine carboxypeptidase, participates in amino acid homeostasis via cleaving amino acids from the C-terminus of a protein substrate. Except the two mentioned previously, some of the other mRNAs listed in Supplementary Table S2 were demonstrated to be associated with lipid and amino acid metabolism. Hierarchical clustering analysis of the 552 differently expressed mRNAs was performed and shown in Figure 2C.

### Altered Ileal Transcriptome Contributed to the Lipid and Amino Acid Metabolism

Normally, lncRNAs exert their functions by interacting with mRNAs under different patterns. To reveal the potential functions of the ileal lncRNAs after DJB, the pathway and Gene Ontology (GO) analysis of aberrantly co-expressed mRNAs were performed. The top 30 significantly enriched pathways were featured and ranked with enrichment scores (Figure 2D). Further analysis indicated that 26 pathways were upregulated and three pathways were downregulated. Apparently, metabolic pathways, including lipid metabolism (12/30), amino acid metabolism (5/30), carbohydrate metabolism (2/30), and energy metabolism (1/30), occupied a respectable majority. Most importantly, robust enrichment was observed in lipid metabolism-related pathways, such as fatty acid degradation, fatty acid elongation, fatty acid biosynthesis, PPAR signaling pathway, biosynthesis of unsaturated fatty acids, and peroxisome as well as in amino acid metabolism-related pathways, such as beta-alanine metabolism, tryptophan metabolism, and valine, leucine, and isoleucine degradation. Overall, the lncRNA-co-expressed mRNA-enriched pathways, which are triggered by the surgery, contribute to the metabolic improvement, especially to lipid and amino acid homeostasis.

Consistent with pathway analysis, the items of “biological processes,” including very long-chain fatty acid catabolic process, negative regulation of fatty acid oxidation, long-chain fatty acid import, and negative regulation of lipoprotein particle clearance, were enriched and involved in the lipid metabolism by manipulating cellular or circulating lipids clearance (Figure 2E). Furthermore, the GO terms for molecular function were correlated with long-chain fatty acid transporter activity, fatty acid transporter activity, and acylglycerol lipase activity, which were essential for lipid metabolism (Figure 2E). Collectively, DJB triggered a shift in the ileal transcriptome somehow, and the altered lncRNAs and co-expressed mRNAs reprogrammed the metabolic systems of the ileum, particularly in the lipid and amino acid metabolism, which finally contributed to the remission of metabolic disorders possibly through increased resting energy expenditure.

### DJB Induced Ileal lncRNA Expression Profiling Shifting

**FIGURE 3:**
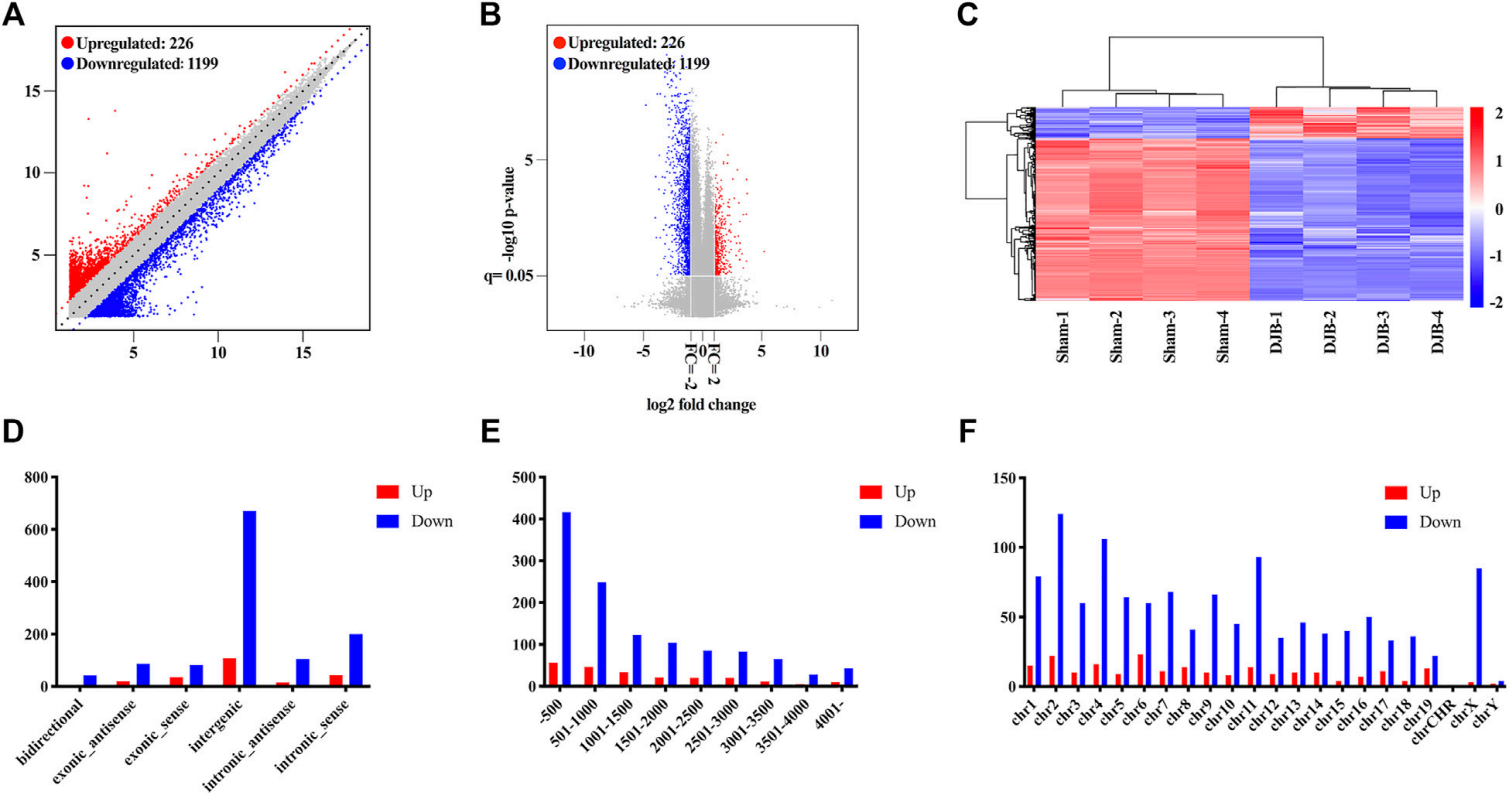
Identification and analysis of distinctively expressed lncRNAs. Scatter plot **(A)** and volcano plot **(B)** of differentially expressed lncRNAs between the DJB and sham groups. **(C)** Heat map of lncRNAs showed hierarchical clustering of differentially expressed lncRNAs in DJB and sham mice (fold change ≥2, *p* < 0.05). Analysis of up- and downregulated lncRNAs based on classification **(D)**, length distribution **(E)**, and chromosome locus **(F)**.

DJB surgery triggered distinctive alteration of ileal lncRNAs’ expression signature with 226 up- and 1,199 downregulated lncRNAs (Figures 3A,B). The 1,425 lncRNAs were further sorted based on the fold change (Supplementary Table S1), and the top 10 up- and downregulated lncRNA are displayed in Supplementary Table S3. The lncRNA *Gas5* with functional annotation, which was reported to play an important role in pancreatic β-cell function and peripheral insulin resistance, was identified in the microarray. Although another lncRNA *Tmem132cos*, named as transmembrane protein 132C (*Tmem132c*) opposite strand, has not been investigated yet, the *Tmem132c* was found correlated negatively with insulin secretion, suggesting that the downregulation of *Tmem132*cos may play a therapeutical role in T2DM remission. Hierarchical clustering indicated that the lncRNA expression patterns between the DJB and sham groups were distinguishable (Figure 3C). In addition, systematic analysis and statistics of differentially expressed ileal lncRNA from different dimensions were carried out and displayed in Figures 3D–F.

### Predicted Target Genes Are Associated With Ileal lncRNAs’ Positive Effect on Metabolic Homeostasis Reconstruction

**FIGURE 4:**
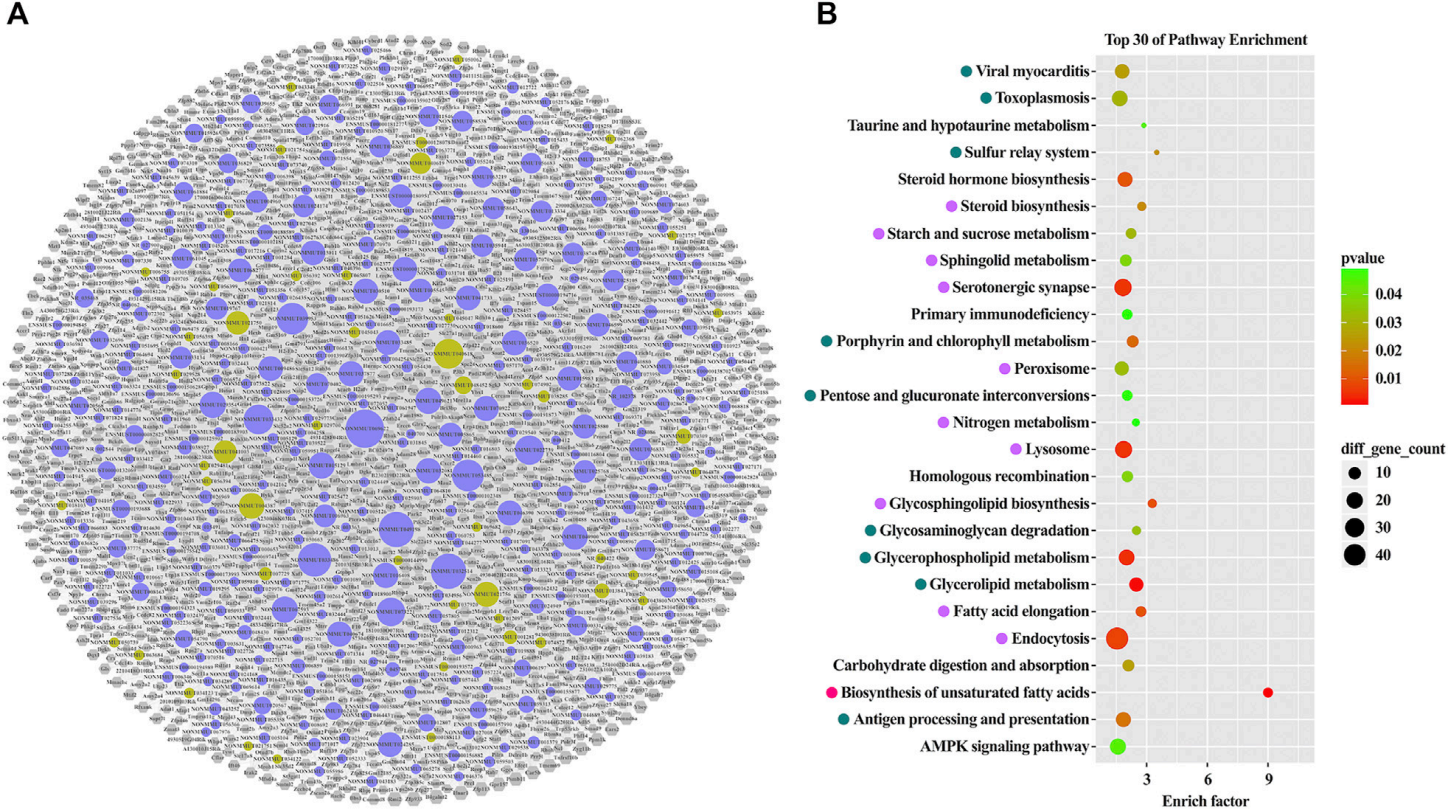
Construction of the lncRNA-target genes interaction network and pathway analysis of the target genes. **(A)** lncRNA-target genes interaction network. Circle nodes with strong yellow and orchid represent up- and downregulated lncRNAs, respectively. Hexagon nodes with gray represent target genes. **(B)** Top 30 significantly enriched pathways ranked with an enrichment score (*p* < 0.05). Trans-, CIS-, and trans and CIS-regulatory pathways are marked with light violet, dark cyan, and pure pink, respectively.

Since lncRNAs exert functions indirectly, prediction of target genes and construction of the interaction network are crucial to identify the most influential ileal lncRNAs after DJB. Utilizing the Cytoscape program, the network was generated with 998 ileal lncRNAs, 2,261 target genes, and 20,876 lncRNA-target gene pairs (Figure 4A). Pathway annotations of predicted target genes indicated that altered ileal lncRNAs may maintain lipid homeostasis by regulating lipid metabolism-related pathways, mainly including biosynthesis of unsaturated fatty acids, fatty acid elongation, AMPK signaling pathway, glycerophospholipid metabolism, and steroid biosynthesis and sphingolipid metabolism (Figure 4B). Furthermore, pathways of taurine and hypotaurine metabolism, sulfur relay system, and nitrogen metabolism, which were associated with the amino acid metabolism, were annotated and beneficial for T2DM amelioration after surgery (Figure 4B, Supplementary Figures S1A–C). Generally, the metabolic benefits from DJB, including the changes in lipid and amino acid metabolism caused by ileal lncRNAs, were comparable between DJB and sham groups.

### Ileal lncRNA–mRNA Pairs Remodeled the Lipid and Amino Acid Metabolism

**FIGURE 5:**
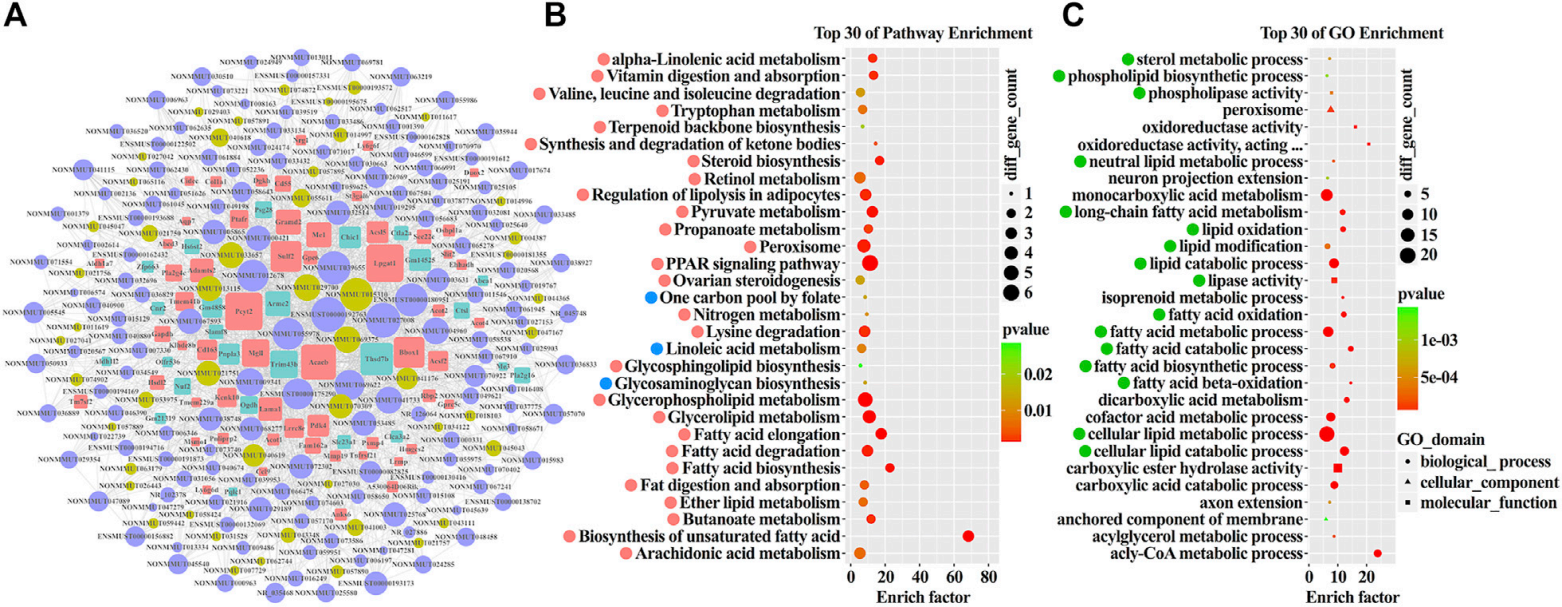
Construction of the lncRNA–mRNA co-expression network and bioinformatic analysis. **(A)** lncRNA–mRNA co-expression network. Circle nodes with strong yellow and orchid represent up- and downregulated lncRNAs, respectively. Square nodes with soft red and slightly desaturated cyan represent up- and downregulated mRNAs, respectively. **(B)** Top 30 significantly enriched pathways ranked with an enrichment score (*p* < 0.05). Up- and downregulated pathways are marked with soft red and soft blue, respectively. **(C)** Top 30 significantly enriched GO items (*p* <0 .05). The important items are marked with green.

In order to screen out the most functional ileal lncRNA–mRNA pairs with potential targeting relationship, the co-expression network was constructed, and bioinformatic analysis was performed. By overlapping the ileal lncRNA-co-expressed mRNAs and predicted target genes, a subgroup containing 82 mRNAs was created. Combining 203 corresponding lncRNAs targeting the 82 mRNAs, 1,459 transcript pairs were generated and finally constituted the network (Figure 5A). The pathways enriched by the subset of lncRNA-co-expressed mRNAs were mostly involved in lipid metabolism, such as fatty acid elongation, fatty acid degradation, fatty acid biosynthesis, biosynthesis of unsaturated fatty acids, PPAR signaling pathway, peroxisome, glycerophospholipid metabolism, and glycerolipid metabolism (Figure 5B, Supplementary Figure S2A). In addition, pathways of valine, leucine, and isoleucine degradation, tryptophan metabolism, lysine degradation, and nitrogen metabolism, which probably acted on postoperative reconstruction of amino acid metabolic homeostasis, were enriched as well (Figure 5B, Supplementary Figures S2A, B). Moreover, the most important GO items, marked with green, referred to lipid biosynthesis, oxidation, and modification (Figure 5C). In short, the subset of ileal mRNAs and their upstream lncRNAs played critical roles in metabolic remission via remodeling the ileal lipid and amino acid metabolism after DJB.

### Identification of Lipid and Amino Acid Metabolism-Related lncRNA–mRNA Pairs

**FIGURE 6:**
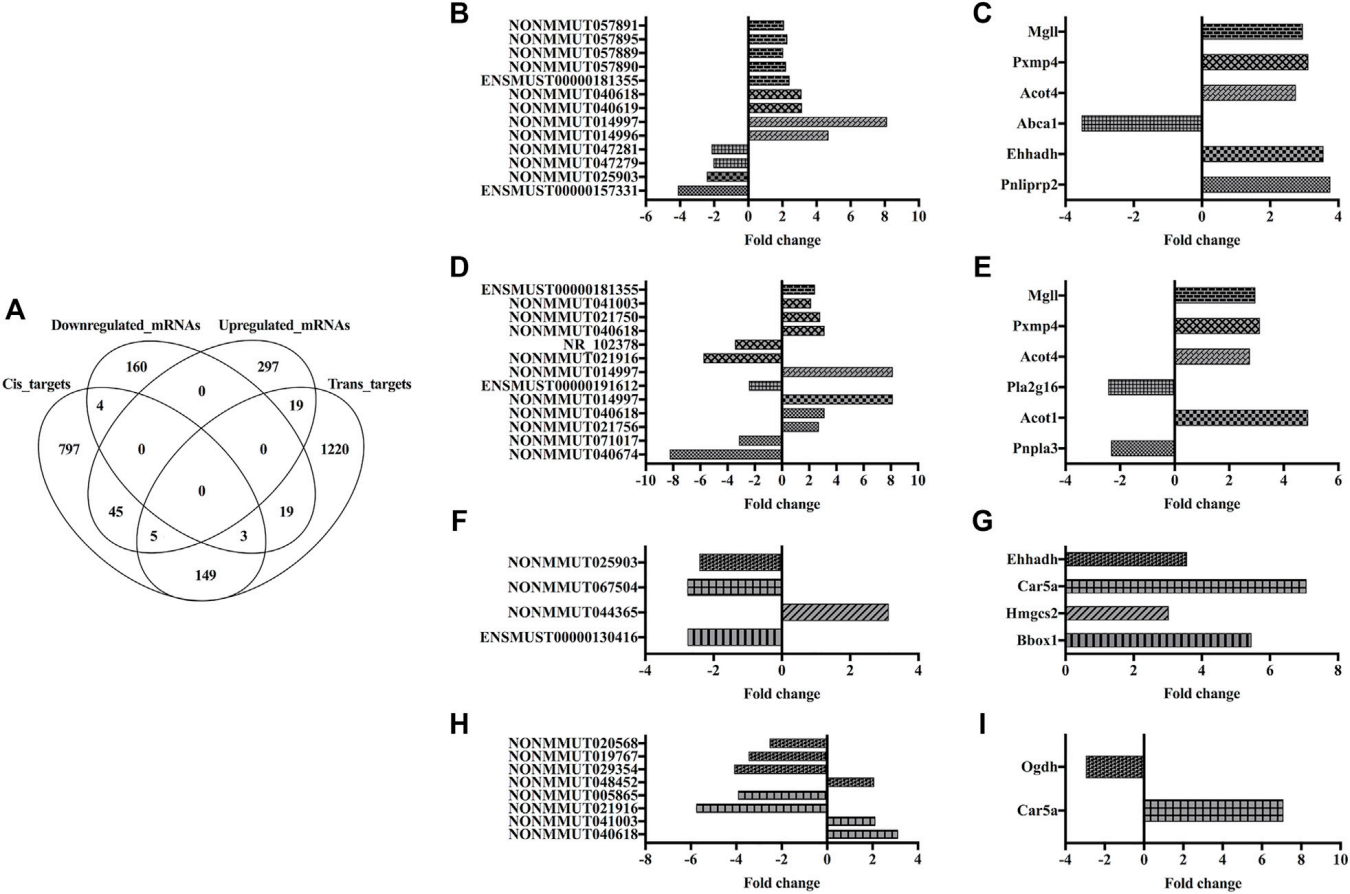
Identification of lipid and amino acid metabolism-related lncRNAs and downstream mRNAs. **(A)** Venn diagram showing the overlapping of up- and downregulated mRNAs with trans- and CIS-targets. Histograms of lipid metabolism pathway-related lncRNA–mRNA pairs via CIS- **(B,C)** or trans- **(D,E)** regulatory patterns. Histograms of amino acid metabolism pathway-related lncRNA–mRNA pairs via CIS- **(F,G)** or trans- **(H,I)** regulatory patterns. The columns with the same fill pattern represent lncRNA–mRNA pairs.

All the aforementioned systematic analyses demonstrated that DJB caused great change of ileal lncRNAs’ expression signature, which further re-established lipid and amino acid metabolic homeostasis of the ileum and participated in the T2DM remission after DJB. Therefore, identification of specific ileal lncRNA–mRNA pairs involved in lipid and amino acid metabolism-related pathways was extremely important. The up- or downregulated mRNAs and CIS- or trans-target genes were input into the Venn program, and 49 CIS-mRNAs, 38 trans-mRNAs, and 8 CIS- and trans-mRNAs were screened out (Figure 6A; Supplementary Table S4). By overlapping the CIS-mRNAs with genes involved in lipid metabolism pathways, 6 CIS-mRNAs, and 13 upstream lncRNAs were verified (Figures 6B,C). In the same way, six trans-mRNAs and 13 lncRNAs were acquired and depicted in Figures 6D,E. Furthermore, amino acid metabolism-related CIS- or trans-mRNAs and relevant lncRNAs are presented in Figures 6F–I, respectively. Notably, the ileal lncRNA NONMMUT040618 and its target mRNAs (*Pxmp4*, *Pnpla3*, and *Car5a*), which were involved in lipid and amino acid metabolism-related pathways, might play vital roles in the remission of metabolic disorders after DJB.

### Ileal Transcription Factors Analysis

**FIGURE 7:**
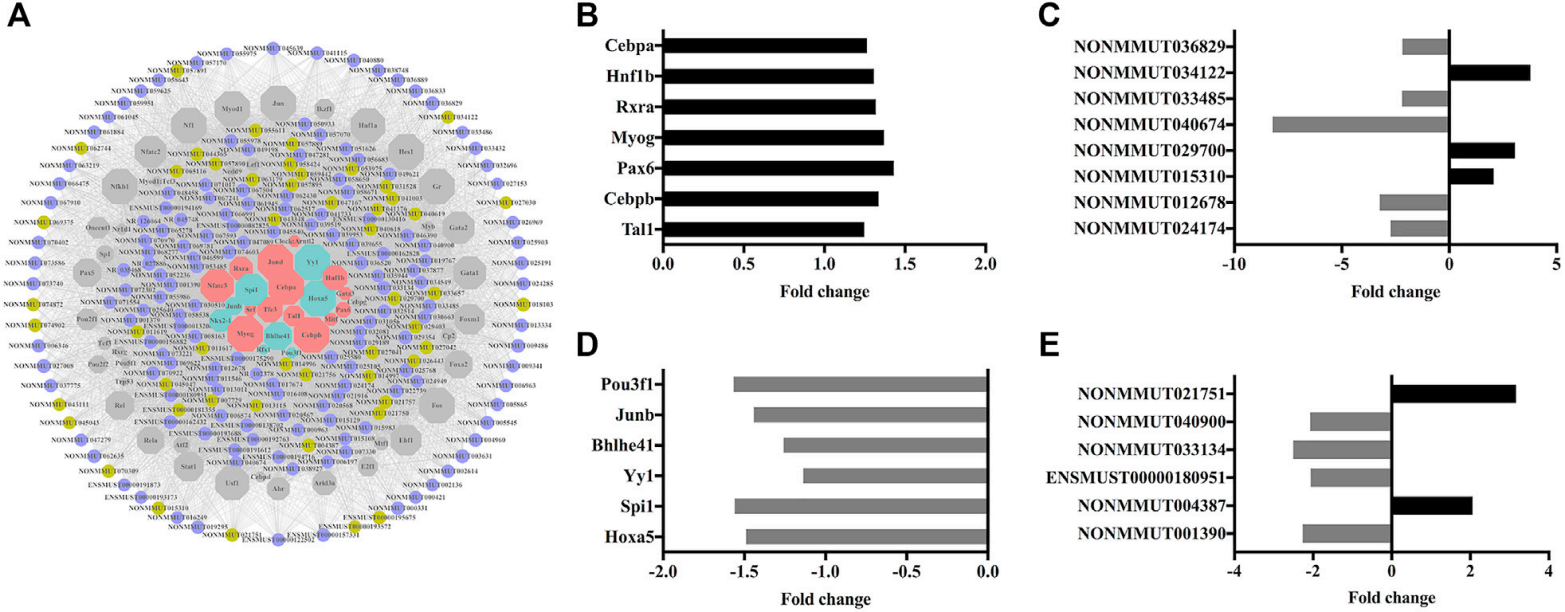
Construction of the lncRNA–TF interaction network and identification of distinctly expressed TFs. **(A)** lncRNA–TF interaction network. Circle nodes with strong yellow and orchid represent up- and downregulated lncRNAs, respectively. Hexagon nodes with soft red, slightly desaturated cyan, and gray represent up-, down-, and unregulated TFs, respectively. Histogram of upregulated TFs **(B)** and their downstream lncRNAs **(C)**. Histogram of downregulated TFs **(D)** and their downstream lncRNAs **(E)**.

To elucidate the upstream regulation network, a group of 63 TFs, targeting 203 ileal lncRNAs involved in the co-expression network, was predicted. All these transcripts and 6,993 lncRNA–TF-targeting pairs constituted the interaction network (Figure 7A). Among the TF group, seven up- and six downregulated TFs were found in the microarray (Figures 7B,D). Moreover, intersecting the distinct subsets of lncRNAs generated by each upregulated TF, eight dysregulated ileal lncRNAs were screened out (Figure 7C, Supplementary Figure S3A). The six downregulated TFs were analyzed with the identical procedure (Figure 7E, Supplementary Figure S3B). Pathway annotation of predicted TFs indicated that the lipid and amino acid metabolism-related pathways were enriched (Supplementary Figure S3C).

### Verification of the Ileal Microarray Data

**FIGURE 8:**
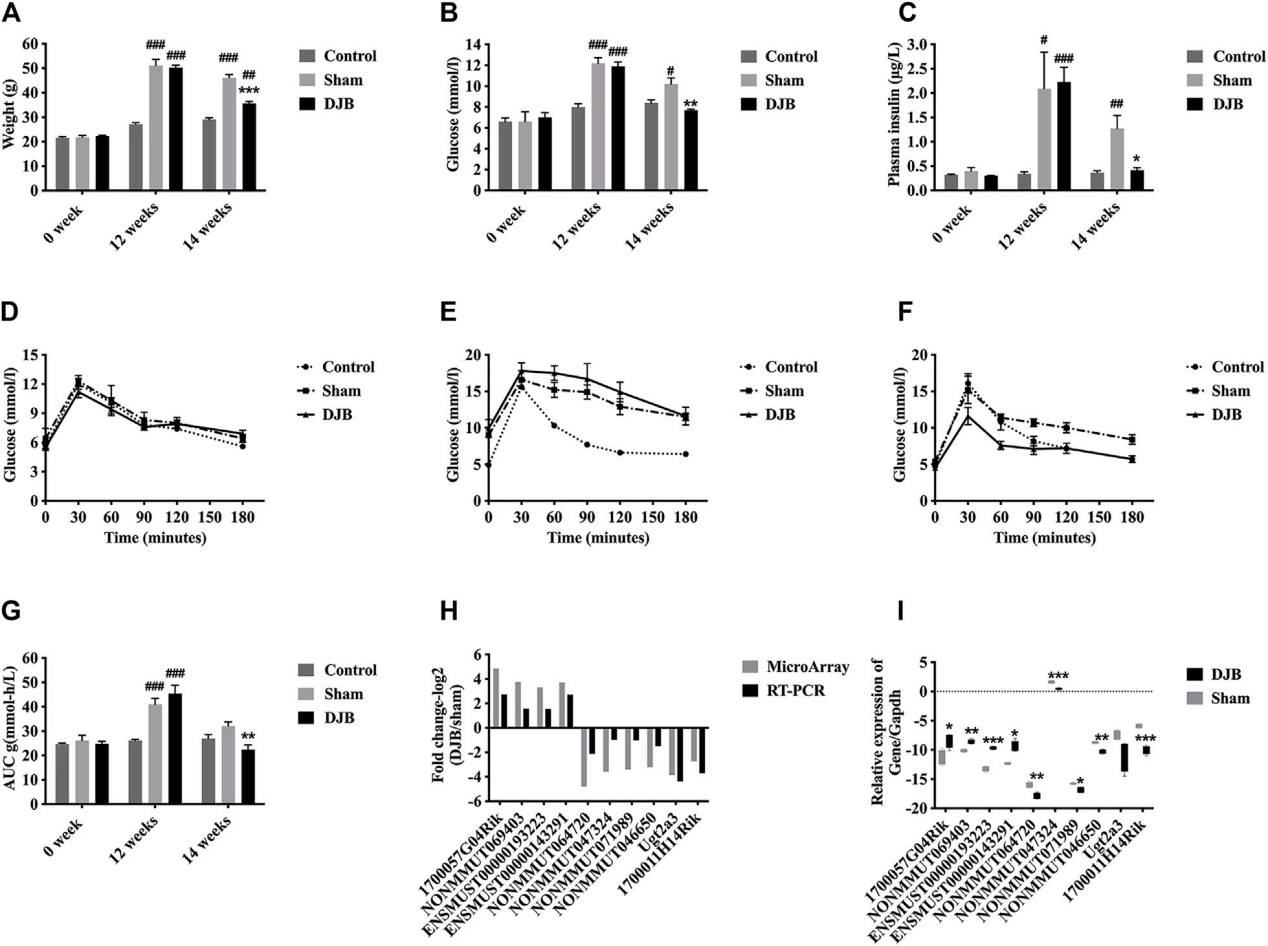
Microarray validation. Weight **(A)**, FBG **(B)**, plasma insulin **(C)**, OGTT **(D)**, week 0 **(E)**, week 12 **(F)**, and week 14 and AUC **(G)** of another cohort of diabetic mice. **(H)** Histograms showing the comparison between microarray data and qRT-PCR results of 10 transcripts. **(I)** Box plot showing the qRT-PCR results of the 10 transcripts with another cohort of mice. All values are shown as mean ± SEM. Statistical differences were analyzed by the Student’s *t*-test. * For DJB group versus sham group, # for DJB or sham group versus control group (*p <0 .05, **p <0 .01, ***p <0 .001, #*p* <0 .05, ##*p* <0 .01, ###*p* <0 .001).

In order to validate the accuracy as well as reliability, 10 dysregulated transcripts in the ileal microarray were randomly selected (Supplementary Table S5), and their expression levels both in samples for microarray and tissues obtained from another cohort of mice undergoing the identical process (Figures 8A–G) were detected using qRT-PCR. The qRT-PCR results from the ileal samples employed in the microarray or newly gained ileal tissues were highly consistent with original microarray data (Figures 8H,I).

## Discussion

DJB induced significant alterations in intestinal lncRNA and mRNA expression patterns. All the dysregulated lncRNAs in the ileum recomposed energy metabolism homeostasis, especially lipid and amino acid metabolism-related pathways. The target genes also indicated that lipid and amino acid metabolism-related pathways were modified by surgical intervention. In addition, the altered TFs, which were responsible for converting surgical information into transcriptome alteration, also served in lipid metabolism- or diabetes-related pathways as expected. These findings imply that the major role of the ileum in MBS is associated with re-establishment of energy homeostasis by alteration of the lipid and amino acid metabolism. Moreover, functional lncRNAs NONMMUT040618 and downstream mRNAs including *Pxmp4*, *Pnpla3*, and *Car5a* were altered by DJB the most, and therefore, are worthy of further investigations.

MBS leads to effective and long-term remission of T2DM as well as other metabolic disorders such as dyslipidemia, hypertension, hypothyroidism, and polycystic ovary syndrome (PCOS) (Pareek et al., 2018; Mintziori et al., 2020). So far, its underlying mechanism is not fully understood. Altered enterohepatic circulation of bile acids (Wahlstrom et al., 2016), gut microbiota, GI hormones, nutritional deficiency, and weight loss (Aron–Wisnewsky and Clement, 2014; Liu et al., 2017), etc., are thought to contribute to the mechanism of MBS. However, none of the aforementioned factors can explain all the consequences of MBS. Rather than studying on a single mechanism, our series of studies focus on the intestinal transcriptome analysis in order to illuminate the functional roles of different small intestinal segments in metabolic regulation after MBS represented by DJB. MBS involves anatomical alteration of the GI tract including size reduction and/or segment bypass. Also as a result, food intake restriction and/or malabsorption as well as increased resting energy expenditure (Das et al., 2003) are induced to cause weight loss. Moreover, it is also the anatomical change that induces beneficial effects in metabolic regulation. We have previously reported that lncRNAs in the duodenum are associated with pancreatic islet secretion and inflammation process, implying that bypass of the duodenum may initiate insulin secretion and attenuate inflammation (Liang et al., 2017), and those in the jejunal Roux limb are associated with gut–brain cross talk and endocrine regulation (Liang et al., 2018). Both are related to the beneficial consequence of MBS including T2DM remission, improvement in lipid metabolism, and endocrine hormonal effects. The present study is the part 3 of this series of studies, which is focused on the role of the ileum.

Ileum, the final segment of the small intestine following the duodenum and jejunum, is primarily responsible for absorption of vitamin B_12_, bile salts, and any nutrients that passed the jejunum under normal circumstances (Matsumoto et al., 2017). In addition to absorption function, cells located in the lining of the ileum secrete protease and carbohydrase enzymes to facilitate protein and carbohydrate digestion (Williams et al., 2011). In addition, we also observed that the whole lipid metabolism networks, especially PPAR signaling pathway, fatty acid elongation, fatty acid degradation, fatty acid biosynthesis, and biosynthesis of unsaturated fatty acids as well as other related pathways, were reprogrammed by the dysregulated ileal lncRNAs after DJB. Thus, MBS may ameliorate the disturbed energy homeostasis via altering lipid metabolism function of the ileum, accelerating energy expenditure.

Ileal amino acid metabolism was another significant function generated by DJB and exerted positive regulation in glycemic control. Under normal physiological conditions, increased plasma amino acid levels after food intake provoke insulin release and mTOR-dependent protein synthesis in muscle (Kimball, 2007). On the cellular level, amino acid homeostasis is maintained by conversion of essential and nonessential amino acids and transfer of amidogen from oxidized amino acids to synthesized amino acids. Once the balance is disrupted, a phenomenon usually preceding or accompanied by dysregulation of glycemia, T2DM could be triggered. For instance, an inappropriate amino acid metabolism results in pancreatic β-cell function disorder and insulin secretion deficiency by disturbing mitochondrial metabolism acutely or altering the insulin granules exocytosis-related genes chronically (Newsholme et al., 2005). Meanwhile, insulin resistance is induced by abnormal plasma levels of essential amino acids and their derivatives (Adams, 2011; Lynch and Adams, 2014). Conversely, improved β-cell function and insulin sensitivity after MBS partially attribute to corrected branched-chain amino acid metabolism and circulation concentrations (Gerszten and Wang, 2011; Magkos et al., 2013). Although the liver is the major site of nitrogen metabolism, surgery-induced amino acid metabolism function in the ileum should be vital for re-establishment of amino acids homeostasis and remission of T2DM.

The limitation of this study is that the screened lncRNAs or mRNAs with potential functions have not been verified yet at the molecular, cellular, and animal levels, which will be carried out in our future studies.

## Conclusion

The present study serves as a fundamental tool for gene analysis after MBS. From this study, we have demonstrated that DJB is able to regulate transcriptional activities of transcriptional factors, which alter lncRNA expression profiles. The target genes of dysregulated lncRNAs in the ileum involve in lipid and amino acid metabolism-related pathways. These findings imply that the role of the ileum in DJB tends to re-establish the energy homeostasis by regulating the lipid and amino acid metabolism. Concluding the present study on the ileum and our previous gene microarray studies on the duodenum and jejunum, different segments of the intestine play different roles in metabolic regulation after MBS.

## Data Availability

The datasets presented in this study can be found in online repositories. The names of the repository/repositories and accession number(s) can be found below: https://www.ncbi.nlm.nih.gov/geo/, GSE190949.
