# Supplementary material for: Alteration of Ileal lncRNAs After Duodenal–Jejunal Bypass Is Associated With Regulation of Lipid and Amino Acid Metabolism
Source: Front Physiol. 2022 Apr 7;13:836918. doi: 10.3389/fphys.2022.836918 (PMC9021573; doi:10.3389/fphys.2022.836918)
Supplement: Supplementary file 1 [file DataSheet1.PDF]

Supplementary Material

TABLE S1 | Numbers of differentially expressed lncRNAs and mRNAs transcripts

		Fold change 2-5	Fold change 5-10	Fold change >10
mRNAs	Up-regulation	321	31	14
	Down-regulation	175	9	2
lncRNAs	Up-regulation	206	14	6
	Down-regulation	1108	83	8

mRNAs = messenger RNAs
lncRNAs = long noncoding RNAs

TABLE S2 | Top 10 upregulated or downregulated mRNAs in the DJB group compared with the sham group

RNA Accession	Gene name	Fold change	*P*-values	Chr	Regulation
NM_001168504	Pla2g4c	159.9500848	0.003269	chr7	Up
NM_153166	Cpne5	47.44559871	0.000843	chr17	Up
NM_001033184	1700057G04Rik	29.40227706	0.028241	chr9	Up
NM_008571	Mcpt2	19.51319965	0.041402	chr14	Up
NM_001198933	Me1	19.03909584	0.000068	chr9	Up
NM_027749	Cpvl	18.08819652	0.000829	chr6	Up
NM_201640	Cyp4a31	14.82257964	0.011149	chr4	Up
NM_010020	Slc6a3	14.72025889	0.000829	chr13	Up
NM_001100181	Cyp4a32	14.18424880	0.009351	chr4	Up
NM_146230	Acaa1b	11.19123306	0.002071	chr9	Up
NM_001048219	Nlrp9a	20.41513136	0.000006	chr7	Down
NM_028094	Ugt2a3	14.50195994	0.006509	chr5	Down
NM_001034856	Btg1-ps2	7.896134441	0.000290	chrX	Down
NM_054074	Defb6	7.335025572	0.003031	chr8	Down
NM_025956	1700011H14Rik	6.615893265	0.000280	chr14	Down
NM_001005425	Zfp663	6.581851869	0.000871	chr2	Down
NM_010776	Mbl2	6.490257067	0.005613	chr19	Down
NM_181407	Me3	5.887722811	0.006818	chr7	Down
NM_134063	Fam208b	5.760339928	0.000074	chr13	Down
NM_029372	Ccdc172	5.215195336	0.001091	chr19	Down

mRNAs = messenger RNAs
DJB = duodenal-jejunal bypass
Chr = chromosomal location

TABLE S3 | Top 10 upregulated or downregulated lncRNAs in the DJB group compared with the sham group

RNA Accession	Gene name	Fold change	*P*-values	Chr	Regulation
NONMMUT039923	NONMMUG024704	37.20103532	0.008286	chr2	Up
NONMMUT069403	NONMMUG042993	13.68659473	0.000119	chr9	Up
ENSMUST00000143291	ENSMUSG00000087404	13.12198776	0.000042	chr11	Up
NONMMUT055566	NONMMUG034489	13.04018996	0.016792	chr6	Up
NONMMUT070309	NONMMUG043513	10.92841445	0.000553	chr9	Up
ENSMUST00000193223	ENSMUSG00000104486	10.05245531	0.009767	chr3	Up
NONMMUT064067	NONMMUG039549	9.024989973	0.006984	chr7	Up
NONMMUT017554	NONMMUG011004	8.905258679	0.014801	chr13	Up
NONMMUT006278	NONMMUG004040	8.890855871	0.004324	chr10	Up
NONMMUT058424	NONMMUG036299	8.870683787	0.000915	chr6	Up
NONMMUT064720	NONMMUG039966	27.82021637	0.000000	chr8	Down
ENSMUST00000116835	ENSMUSG00000080485	15.73497442	0.000034	chr16	Down
NONMMUT030361	NONMMUG018770	14.2643026	0.000000	chr17	Down
NONMMUT046523	NONMMUG028743	12.92587746	0.000087	chr4	Down
NONMMUT009001	NONMMUG005579	12.72412991	0.000000	chr11	Down
NONMMUT009936	NONMMUG006231	12.61438143	0.000007	chr11	Down
NONMMUT047324	NONMMUG029288	11.96553955	0.000000	chr4	Down
NONMMUT071989	NA	10.51560564	0.000035	chrX	Down
NONMMUT070885	NONMMUG043899	9.649086615	0.000118	chr9	Down
NR_110489	AW822252	9.624018272	0.013708	chrX	Down

lncRNAs = long noncoding RNAs
DJB = duodenal-jejunal bypass
Chr = chromosomal location
NA = not applicable

TABLE S4 | Intersection of predicted targets and lncRNAs-coexpressed mRNAs

Cis-mRNAs Gene Symbol			Cis- & trans-mRNAs Gene Symbol	Trans-mRNAs Gene Symbol		
Abca1	Ehhadh	Osbpl1a	Acot4	A530064D06Rik	Ctsl	Ctsl
Abcd3	Fam162a	Paqr8	Car5a	Acot1	Efr3b	Olfr536
Acacb	Gapdh	Pcyt2	Clca3a1	Acot2	Gm14525	Pla2g16
Acsl5	Gpc6	Pdk4	Ctla2a	Acsf2	Gm21319	Pla2g4c
Aldh1a7	Gramd2	Pnliprp2	Me3	Adamts2	Gm4858	Pnpla3
Aldh1l2	Hmgcs2	Pqlc1	Mgll	Aqp7	Gprc5c	Psg28
Anks6	Kcnk10	Rab30	Pxmp4	Armc2	Hs6st2	Ptafr
Bbox1	Klhdc8b	Rbp2	Tmem229a	Bco1	Hsdl2	Sec22c
Cd300lb	Lama1	Slit2		Ccl9	Il1rl1	Slamf8
Cd55	Lpgat1	St3gal6		Cd163	Lrrc8e	Slc23a1
Cidec	Lrmp	Sulf2		Chic1	Mmp19	Trim43b
Col1a1	Ly6g6d	Thsd7b		Clca3a2	Nuf2	Zfp663
Cyp4a31	Ly6g6f	Tm7sf2		Cnr2	Ociad2	
Dclk1	Me1	Tmem41b				
Dgkh	Msmo1	Tnfrsf21				
Dkk4	Nrg1	Tppp				
Duox2						

mRNAs = messenger RNAs

Cis = cis-regulatory pattern

Trans = trans-regulatory pattern

Cis-mRNAs = intersection of cis-targets and lncRNA-coexpressed mRNAs

Trans-mRNAs = intersection of trans-targets and lncRNA-coexpressed mRNAs

TABLE S5 | The lncRNAs and mRNAs primers used in quantitative real-time PCR

Primer Name	Sequence (5' to 3')
1700057G04Rik-F	GGCAGGATTTCCAAGCACT
1700057G04Rik-R	CGAGGTCTAAAGGGAACTGG
NONMMUT069403-F	GCTCGTCTTTCCTCTGTCGT
NONMMUT069403-R	CTGGCCGGTGATCTTATGAA
ENSMUST00000193223-F	GTCTTACGTGCAGCCACAAT
ENSMUST00000193223-R	CACACCTTATTACACAAACTTCTGG
ENSMUST00000143291-F	CTGTGCAATCGGAAAGAAAAG
ENSMUST00000143291-R	AGGGAGGTTTTTGTGAGCTGT
NONMMUT064720-F	TGGGAACTATAATGGCTTTGG
NONMMUT064720-R	TGAGCTTCTTCCACAAACCTG
NONMMUT047324-F	GGCCTTTTATGACTGGAGCTG
NONMMUT047324-R	CAGGAGAGCAACCATACCATC
NONMMUT071989-F	TCAAGAACAACAAGGGTAATGCT
NONMMUT071989-R	GCCTTAGCTTCTCTGCCTTCT
NONMMUT046650-F	TGAGCGTGGTGCTGGAGTT
NONMMUT046650-R	GAAGCAGTAGGCCAGACAACACT
Ugt2a3-F	CCTCACAACATCGCTCACAT
Ugt2a3-R	TGCTCGTCATTGTACTGATGC
1700011H14Rik-F	CTGGTGAGGACCAAGACAGAG
1700011H14Rik-R	CTGGGGTAGCGTCTAGCTTTC
Mus Gapdh-F	CACAATTTCCATCCCAGACC
Mus Gapdh-R	GTGGGTGCAGCGAACTTTAT

lncRNAs = long noncoding RNAs
mRNAs = messenger RNAs
PCR = polymerase chain reaction
F = forward
R = reverse
A = adenine
T = thymine
C = cytosine
G = guanine

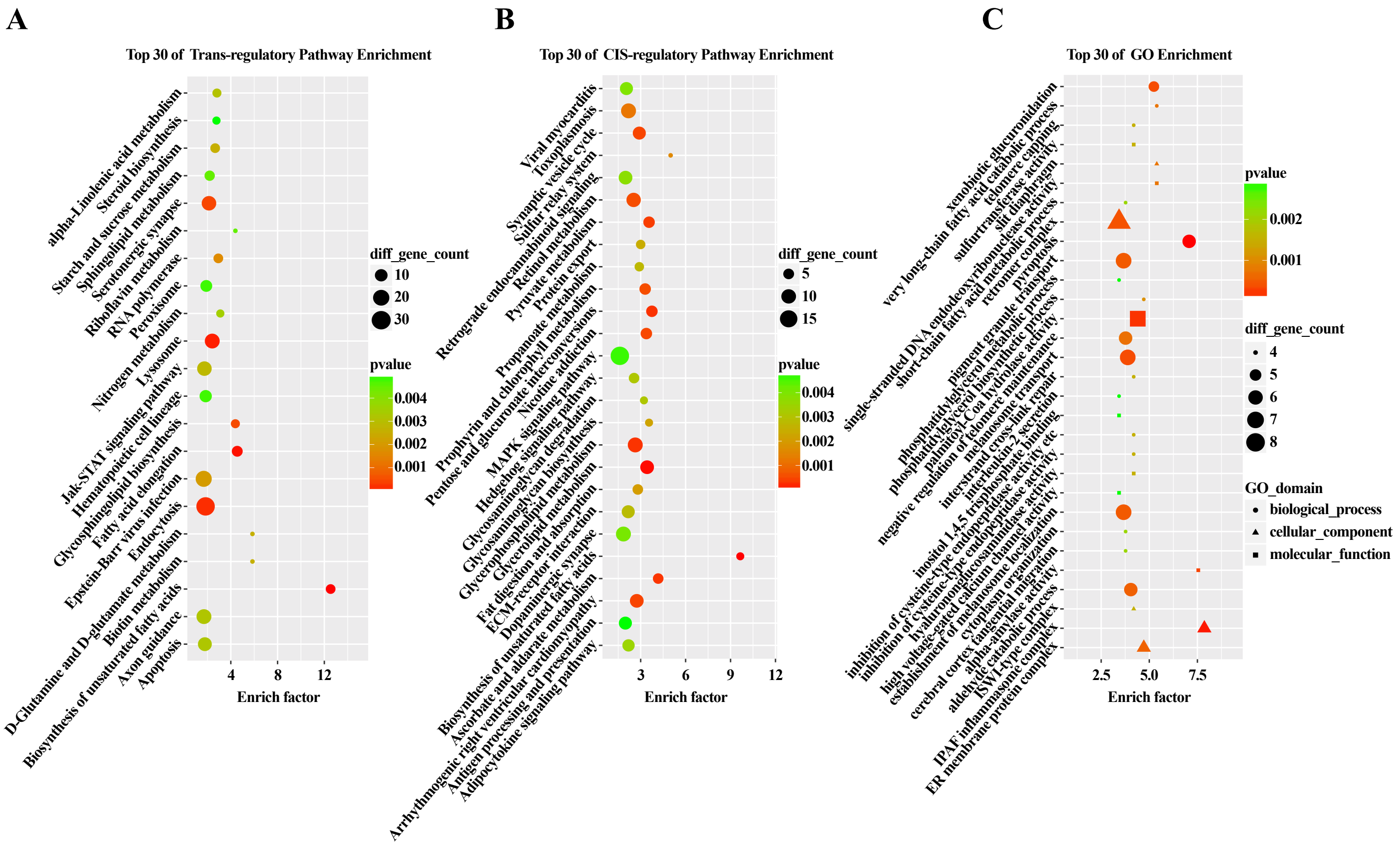

Figure S1 | The top 30 trans- (A) and CIS- (B) regulatory pathways ranked with enrichment score (P < 0.05). (C) The top 30 significantly enriched GO items (P < 0.05).

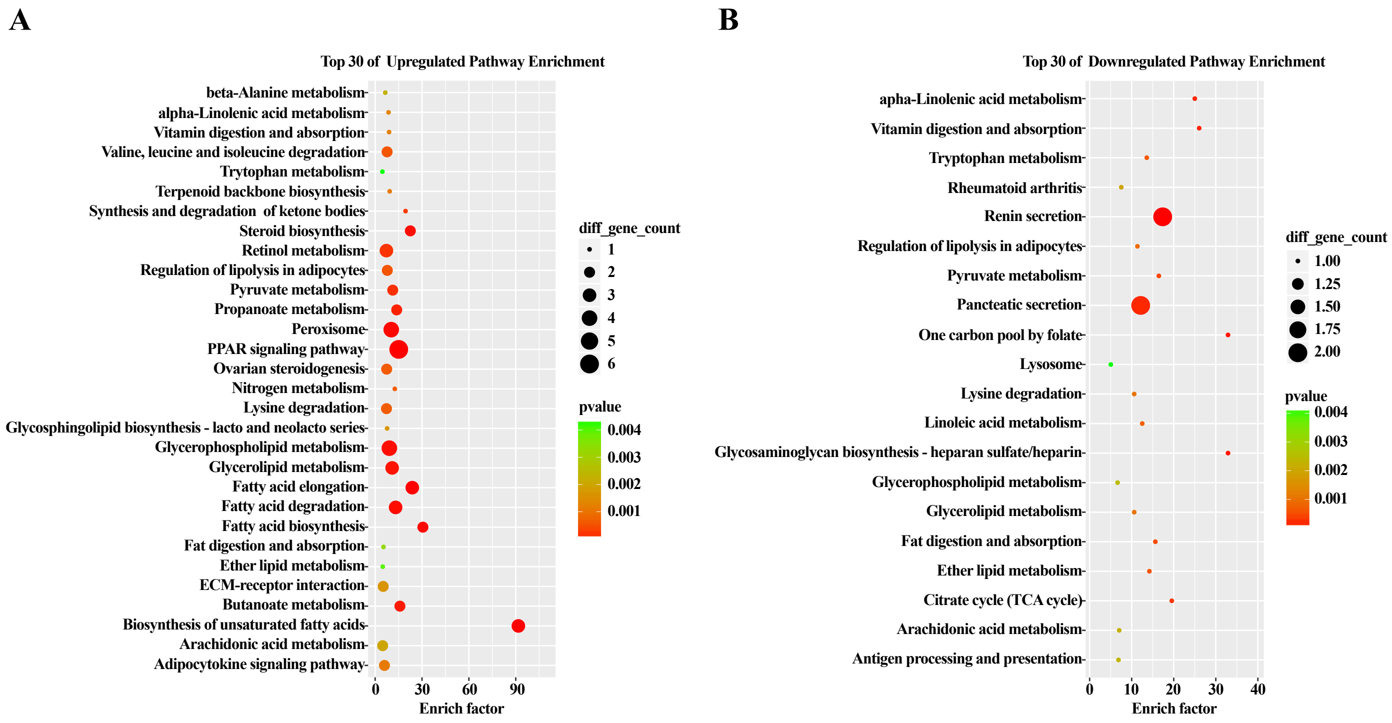

Figure S2 | The top 30 up- (A) and down-regulated (B) pathways ranked with enrichment score ($P < 0.05$).

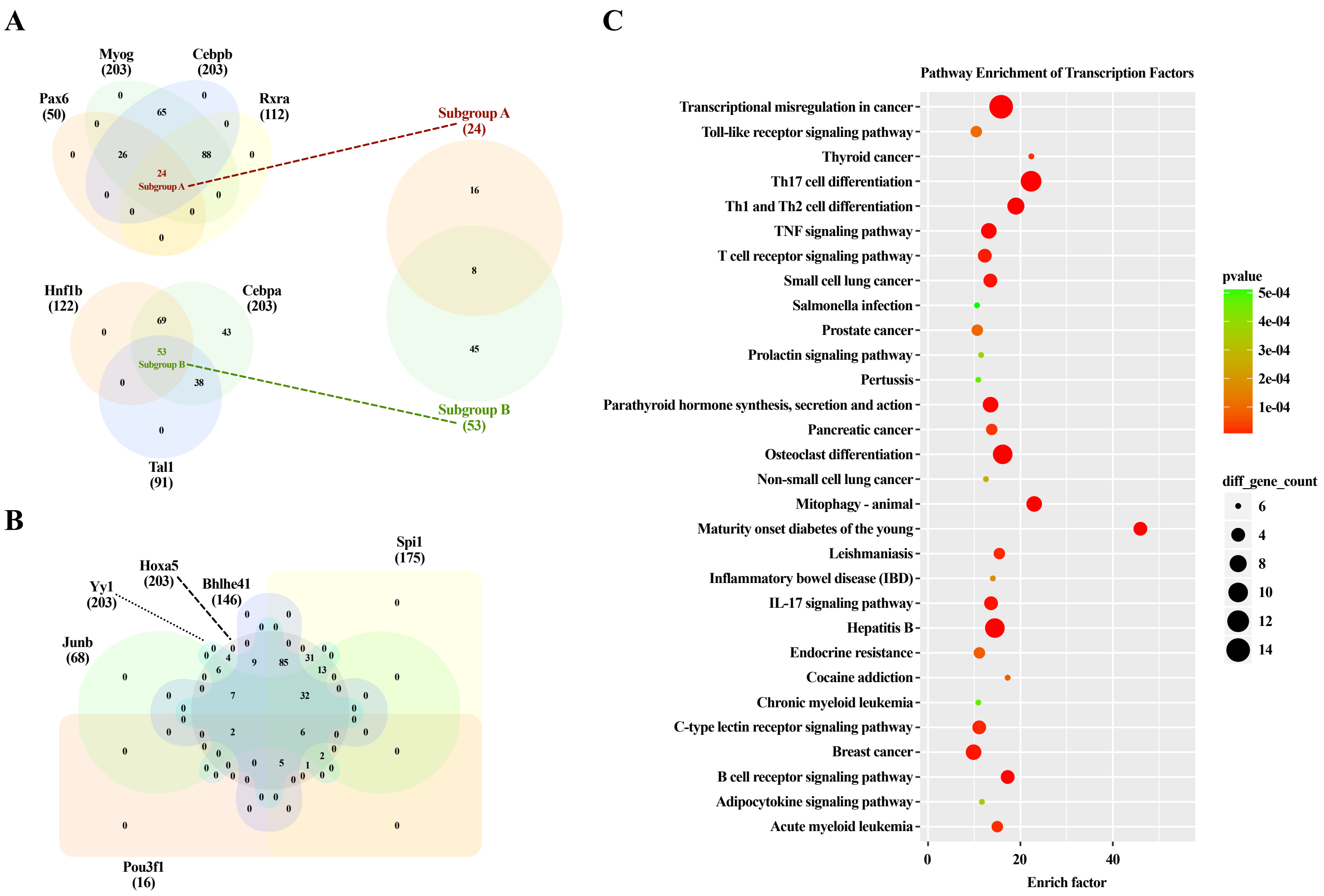

Figure S3 | Venn diagram showing the overlap between different subsets of lncRNAs generated by up- (A) and down-regulated TFs (B). (C) The pathways enriched by TFs ($P < 0.05$).
